# Potential cost-effectiveness of outdoor unhealthy food and drink advertising restrictions in Western Australia

**DOI:** 10.1017/S1368980026102249

**Published:** 2026-02-26

**Authors:** Jaithri Ananthapavan, Mary Rose Angeles, Emma Groves, Victoria Brown, Kathryn Backholer, Gary Sacks, Ainslie Sartori

**Affiliations:** 1 Deakin University, Faculty of Health, School of Health and Social Development, Institute for Health Transformation, Deakin Health Economics, Geelong, Australia; 2 Deakin University, Faculty of Health, School of Health and Social Development, Institute for Health Transformation, Global Centre for Preventive Health and Nutrition, Geelong, Australia; 3 Cancer Council Western Australia, Subiaco, Australia

**Keywords:** Outdoor advertising, Food marketing restrictions, Food policy, Cost-effectiveness, Economic evaluation, Cost analysis

## Abstract

**Objective:**

To model the potential value for money of implementing proposed unhealthy food advertising restrictions on Western Australian (WA) transport-owned assets to prevent obesity-related diseases.

**Design:**

A cost–benefit analysis using a societal perspective was undertaken to model the policy intervention over a 30-year time horizon. The effectiveness of the intervention was based on a similar policy implemented in the United Kingdom by Transport for London, adapted to the WA context. The ACE-Obesity Policy model, a validated multi-state lifetable Markov model, was used to assess the expected health (quantified as health-adjusted life years (HALY)) and economic outcomes of the intervention’s impact on unhealthy food consumption. The potential costs of policy development and monitoring and revenue impacts on government and industry (outdoor advertising companies) were included in the modelled analysis.

**Setting:**

Western Australia.

**Participants:**

Greater Perth population.

**Results:**

The cost of implementing the policy was estimated at A$28 million (95 % uncertainty intervals (UI): $23, $35), 71 % borne by the government and the remaining by outdoor advertisers. A mean population weight reduction of 0·58 kg (95 % UI: 0·28, 0·90) was estimated, which translated to 5906 health-adjusted life years gained (95 % UI: 2750, 9084) with a monetary value of A$1374 million (95 % UI: $642, $2112). Eight percent of the monetised benefits were attributed to healthcare cost savings, while 92 % were associated with monetised health gains. The intervention was estimated to generate a net-present value of $1346 million (95 % UI: $614, $2082) and benefit–cost ratio of 50 (95 % UI: 23, 81).

**Conclusion:**

Policy to restrict advertising of unhealthy foods on WA transport-owned assets is likely to represent excellent value for money.

The global burden of disease attributable to overweight and obesity continues to rise, and in Australia, it has recently overtaken tobacco use as the leading risk factor for morbidity and mortality^(1)^. Excess weight also imposes a significant financial burden on both the Australian healthcare system and broader society, with an estimated annual cost of approximately A$39 billion^(2)^. The ubiquitous marketing of unhealthy foods (high in salt, added sugar and/or saturated fat) across various media platforms impacts dietary choices, and there is credible evidence of it being causally linked to consumption^(3)^. The WHO has highlighted that unhealthy food marketing is a threat to public health and recommends governments implement comprehensive restrictions^(4)^.

Out of home (OOH) advertising consists of various formats including roadside billboards, public transport assets and other public spaces such as sports stadiums and shopping centres. This form of advertising continues to expand in Australia as an effective form of marketing to large and unrestricted audiences, with approximately 93 % of Australians living in and around state capital cities being exposed to outdoor media assets daily^(5)^.

Australian audits have shown that there is a high prevalence of unhealthy foods being advertised on OOH assets^(6)^, and many of these assets are government owned (e.g. trains, buses and roadside billboards). Given the negative impact of unhealthy food advertising on health and the incongruence between unhealthy food advertising on government assets and government imperatives and strategies to improve public health, the public health community in Australia has called for the removal of unhealthy food advertising on government-owned assets^(7,8)^.

A recent scoping review identified nine jurisdictions that have implemented policies to restrict unhealthy food advertising on publicly owned assets^(9)^. However, there is limited evaluation of these policies to demonstrate their effectiveness on population dietary behaviours. The exception is the Transport for London (TfL) policy, which imposed restrictions on unhealthy food advertising on TfL assets in 2019. A controlled interrupted time series analysis of the TfL policy showed that it was associated with relative reductions in take-home purchases of unhealthy foods^(10)^.

In addition to the evidence of policy effectiveness, Australian governments require evidence on the value for money of policies prior to them being considered for implementation^(11)^. Consideration of the costs and benefits of a proposed policy can inform the efficient allocation of scarce societal resources. An economic modelling study of the TfL policy showed that OOH unhealthy food and drink advertising restrictions were likely to result in significant health gains, healthcare cost savings and have a positive equity impact given the socio-economic patterning of obesity prevalence^(12)^. However, this study did not investigate the cost of policy implementation or its impact on industry. As a result, the overall costs and benefits of the policy from a societal perspective remain unknown^(12)^.

In 2021, the Western Australian (WA) Government made an election commitment to form a working group to investigate the feasibility of a policy to remove unhealthy food advertising from State-owned assets^(13)^; however, the policy is yet to be implemented^(14)^. The aim of this study was to inform policy implementation by undertaking cost–benefit analyses using a societal perspective to assess the potential value for money of a policy to restrict unhealthy food and drink advertisements on WA State-owned public transport assets.

## Methods

### The proposed policy

The proposed intervention involves developing and implementing a state government policy to remove all unhealthy food and drink advertising from WA State government-owned public transport assets. These include assets owned by the Public Transport Authority (PTA), comprising buses, trains and train stations; and those owned by Main Roads WA, including billboards that are visible from state roads and bridges. Public transport media assets that would be excluded from this policy include bus shelters and roadside seats, which are owned by local governments, and sports stadiums, which have complex ownership and governance agreements for advertising rights.

The policy defines unhealthy food using the Council of Australian Governments guidelines on foods and drinks that are not recommended for promotion to reduce children’s exposure to unhealthy foods^(15)^. The Council of Australian Governments food category-based guideline is aligned with the Australian Dietary Guidelines, easy to implement and able to better identify unhealthy food products compared with other nutrient profiling models^(15)^. To ensure comprehensiveness, the modelled policy also restricts master brand advertising associated with unhealthy food and drink. Master brand advertising promotes the company’s brand name and aims to build recognition and trust in the company and therefore can influence consumer preferences and purchase intent, even when specific unhealthy products are not advertised^(16,17)^.

### Overview of the economic evaluation

Cost–benefit analysis is a type of economic evaluation where the costs and benefits of proposed government actions are monetised to assess whether the benefit to society justifies the expenditure of limited resources. Although not commonly used for health sector economic evaluations, it is the preferred economic analysis to inform WA government decision-making by the Treasury and Transport Departments^(18)^. The incremental costs and benefits of the policy were estimated from a societal perspective using a status quo (no policy) comparator. Consistent with several government guidance documents, the time horizon for the analysis was 30 years (with different time horizons tested in sensitivity analyses (Scenario 1–2)) and a discount rate of 7 %^(11)^ was applied to both costs and benefits and reported for the 2019 reference year. Costs were adjusted to 2019 values using the consumer price index^(19)^. The results of the analyses were presented as net present value and benefit cost ratios (BCR). A positive net present value indicates that the intervention generates net societal benefits with its magnitude reflecting the size of net benefits. A BCR greater than 1 demonstrates that benefits outweigh costs with higher values indicating greater return on investment.

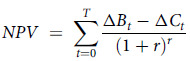




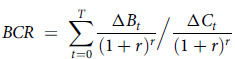

where


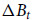
 = Intervention net monetary benefits in year *t*. All impacts of the intervention were included on the ‘benefits’ side of the equation


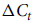
 = Intervention net costs in year *t*

*r* = Real social discount rate (7 % in primary analysis)

*T* = Number of years in the analysis period (30 years for the primary analysis)

### Policy impact on population weight and body mass index

**Figure 1.**
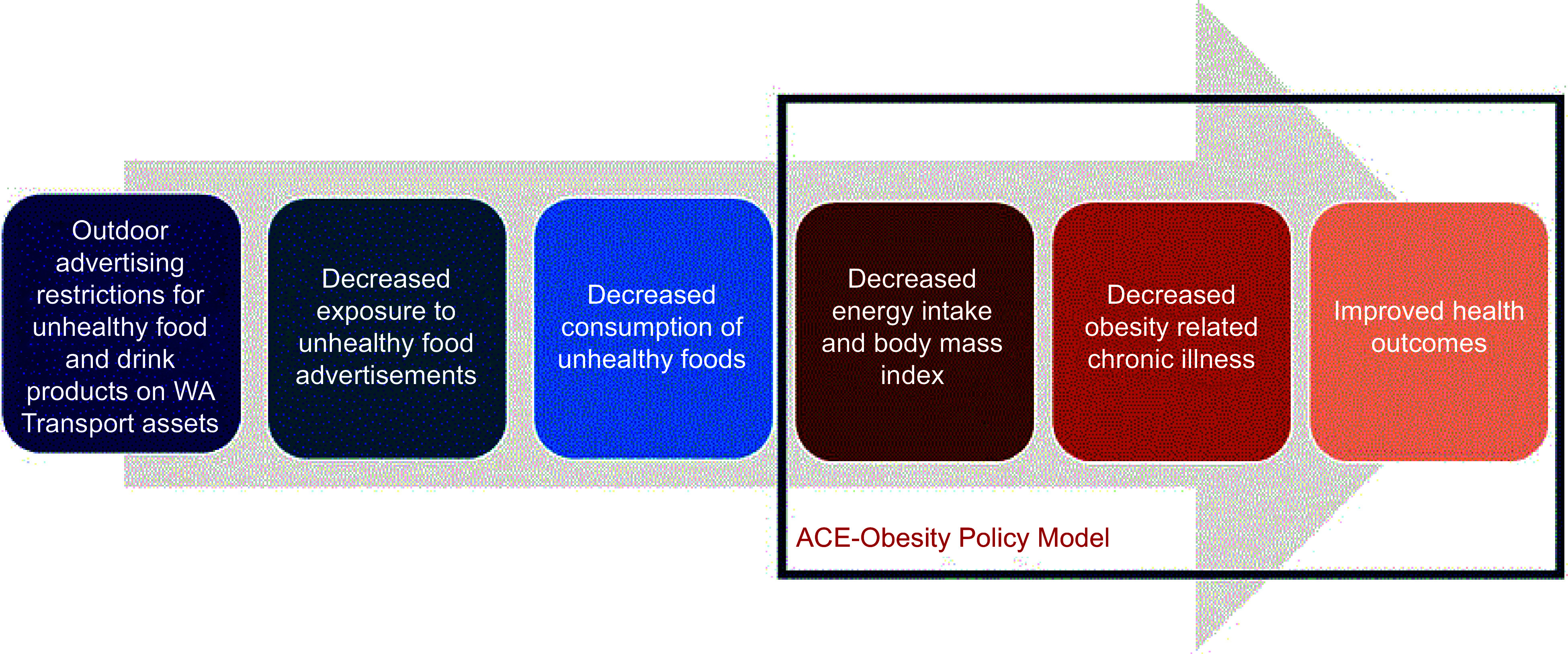
Intervention logic pathway.

The policy was modelled to impact the population of Greater Perth (the capital city of Western Australia), who are most likely to be exposed to advertising on public transport assets^(5)^. The intervention logic pathway is shown in Figure 1. The effectiveness of the policy was based on the evaluation of the TfL policy by Yau *et al.* ^(10)^ The controlled interrupted time series analysis of 1970 households (in London (intervention) and North of England (control)) used panel data to estimate the energy and nutrients from unhealthy food and drink products in average weekly household grocery purchases in the post-intervention period (44 weeks) compared with a counterfactual constructed from the control and pre-intervention (36 weeks) purchasing data^(10)^. The study found that there was a relative change of −6·7 % (95 % CI: −10·1, −3·2) in weekly household energy purchased from foods high in fat, salt and sugar in the intervention group compared with the control group post policy implementation.

#### Effectiveness adjustments

The overall change in unhealthy food purchasing from the TfL analysis was adjusted to reflect the key differences in the TfL and the proposed WA policy and exposure to OOH advertising in London compared to Perth:

- The TfL policy covers more transport assets compared with the proposed WA policy. The TfL policy applies to 90 % of bus shelters within the Greater London Authority administrative area,^(20)^ whereas the WA policy does not apply to any bus shelters in Perth. However, the proposed intervention is more comprehensive than TfL as there are no exemptions and restricts all master branding, whereas the TfL policy allows master branding alongside healthy products. Given that these policy provisions have positive and negative effects on the impact of the policy on unhealthy food advertising exposure and therefore consumption, for the primary analysis, it was assumed that no adjustments to the TfL effect size were required. In sensitivity analyses (Scenario 3), a further 18 % reduction in the effectiveness of the TfL policy was applied to account for the assumed reduced coverage of public transport assets for WA compared with the TfL policy. This was calculated based on the assumption that one-fifth of media assets in WA are bus shelters (others being buses, roadside billboards, trains, train stations and bridges), and exposure to OOH advertising is equivalent across these assets.
- Although high quality and comparable evidence on OOH advertising exposure across the two contexts (Perth and London) is lacking, the limited evidence from industry reports state that 98 % of the UK population are exposed to OOH advertisements each week^(21)^, while the corresponding value for Australians is 78 %^(22)^, which increases to 93 % of the population living in Australian state capital cities (including Perth) being exposed daily^(5)^. For the primary analysis, we assumed a 20 % reduction in effectiveness from the TfL analysis but also tested the assumption of no difference in effectiveness between the two policies in sensitivity analyses (Scenario 4).

The adjustments made to the effect size in the primary and sensitivity analyses were discussed with the project advisory group, which consisted of food policy academic experts, representatives from public health NGOs and policymakers. Table 1 shows key parameter values used in the primary analysis. Table 2 shows the various sensitivity analyses undertaken including scenarios where the effect size estimate was varied.

**Table 1.** Key parameters used in the primary cost–benefit analysis

Key parameters	Values used in primary analysis and uncertainty distribution (cost in 2019)	Sources and comments
Intervention effectiveness – percentage change in weekly household mean energy intake.	–5·36 % (95 % UI: –2·64, –8·14) Normal distribution	20 % reduction to Yau *et al.* ^(10)^ estimate to account for differences in population exposure to OOH advertisements.
Food categories included in effectiveness estimation	Chocolate confectionery, sugar confectionery and sweet spreads (e.g. jams and chocolate spreads) Biscuits, cakes, puddings, ice cream, custard, ready-to-eat icing, jellies and pastries (e.g. sweet snacks, desserts, ice creams and ice confections) Sugar-sweetened drinks (e.g. soft drinks and flavoured mineral waters, energy and sport drinks, cordials, fruit/vegetable drinks with added sugar and slushies) Savoury snacks (e.g. savoury and/or flavoured crisps (potato/corn/grain/vegetables) Fast food (e.g. burgers, pizza, nachos, hot chips, wedges, hashbrowns) Mean consumption of each of the above food categories were estimated by 5-year age and sex group for the Greater Perth population. Lognormal distribution	Perth population consumption from ABS data^(23,24)^. Food categories and foods included in each food category based on COAG guidelines^*^.
Estimated cost to government to develop and implement the policy	A$755 915 (95 % UI: $533 455, $1 011 806) Unit costs, time spent developing the policy and the number of FTE required for this task were varied using a Pert distribution using mean ± 25 %	2·4 FTE for 12 months – based on advice from WA policymakers. Unit cost A$163 per hour in 2021/22 values^(52)^ adjusted to 2019 values
Estimated revenue loss to PTA and Main Roads	Total revenue loss: A$1 381 748 per year (95 % UI: $994 945, $1 899 238) PTA: A$562 556 per year (95 % UI: $462 329, $659 818) Main roads WA: A$819 192 per year (95 % UI: $ 493 634, $1 282 192) The proportion of food advertising that is for unhealthy foods and Main roads WA share of advertising revenue was varied using a Pert distribution with a mean ±25 %.	PTA revenue from food and drink advertising^(39)^: 2017/18 A$1 006 050 and 2018/19 A$1 002 984 Proportion of food advertising that is for unhealthy foods: 55 %^(6,53)^ Main roads WA share of advertising revenue: 58 %^(40)^
Estimated cost to government to monitor the policy	A$73 060 per year (95 % UI: $59 226, $86 832) Weekly wage of personnel was varied using a Gamma distribution. Salary on-cost, annual leave loading and the number of FTE required for this task were varied using a Pert distribution using mean ±25 %.	Weekly wage of personnel for policy monitoring: $1258 in 2021 values adjusted to 2019 values^(54)^ Labour on-cost (14 % salary cost^(36,54)^): $8849 in 2019 values Annual leave loading (17·5 % weekly salary cost, 4 weeks per annum^(37)^): $851 in 2019 values Assumption: 1·0 FTE administration and compliance officer.
Cost of early termination of contracts	Not included	There are no data on the cost of early contract termination.
Potential impact on OOH advertising industry profits	A$596 313 per year (95 % UI: $494 034, $704 306) The proportion of discretionary ads over all ads was varied using a Pert distribution using mean ±25 %.	APN revenue from transport assets: A$302·3M in 2017 values^(40)^ adjusted to 2019 values and adjusted by advertising concentration in WA (7·2 %^(42)^), proportion of discretionary ads over all ads (21 %^(6,53)^) and a 13 % profit margin^(43)^.
Monetised health gains	A$213 000 per HALY No uncertainty included. Value tested in sensitivity analyses.	Incremental HALY gain estimated using the ACE-Obesity Policy model^(28,29)^. VSLY^(33)^ applied to each HALY gained.

A$, Australian dollars in 2019 values; ABS, Australian Bureau of Statistics; CBA, cost-benefit analysis; FTE, full time equivalent; HALY, Health-adjusted life years; M, millions; OOH, out of home; PTA, Public Transport Authority; UI, uncertainty interval; VSLY, value of a statistical life year; WA, Western Australia.

* COAG (Council of Australian Governments) Health Council, 2018. National interim guide to reduce children’s exposure to unhealthy food and drink promotion – 2018 (Guideline). Available at: https://www.health.gov.au/resources/publications/national-interim-guide-to-reduce-childrens-exposure-to-unhealthy-food-and-drink-promotion-2018?language=en

**Table 2.** Description of scenario analyses

Parameter	Scenario no.	Parameter value	Justification
Modelling parameters	Scenario 1	10-year time horizon	Project advisory group stated that this is a policy relevant time horizon.
	Scenario 2	4-year time horizon	Project advisory group stated that this is a policy relevant time horizon. It aligns with Australian State government election cycles.
Effectiveness	Scenario 3	Effect size applied to all unhealthy food products: –4·2 % (95 % UI: –2·1, –6·3)	A further 18 % reduction based on differences in public transport asset policy coverage between TfL^(20)^ and WA.
	Scenario 4	Effect size applied to all unhealthy food products: –6·7 % (95 % UI: –10·1, –3·2)	No adjustment made to TfL policy effectiveness^(10)^ as OOH advertising exposure in the UK^(21,55)^ is similar to Australian state capital cities^(5)^.
	Scenario 5	Effect size: Chocolate & Confectionary: –15·6 % (95 % UI: –11·0 %, –20·4 %) Puddings & Biscuits: –5·2 % (95 % UI: 0·4 %, 10·0 %)	Applying TfL statistically significant food categories only.
	Scenario 6	VSLY valued at A$314 772 and lifetime time horizon	A more contemporary estimate of the VSLY^(11,34)^ and using the recommended time horizon for preventive health CBA
Costs	Scenario 7	Cost of legislation in 2019: $1 302 830 (95 % UI: $1 129 184, $1 494 272)^(38)^	Although it is most likely that this intervention will be developed as a policy, this scenario tested if legislation were required, which would result in increased costs.
	Scenario 8	No change in government and advertiser advertising revenue	Based on evidence of no loss of advertising revenue after the TfL policy was implemented^(12,56)^.
	Scenario 9	Government (PTA and Main Roads WA) and advertiser revenue loss: year 1: 100 %, year 2: 75 %, year 3: 50 %, year 4: 25 %, year 5 and onwards 0 %	Accounts for potential for advertising replacement on outdoor assets over time.
Costs and effectiveness	Scenario 10	Phase in the intervention in 10 years when the contract ends.	Demonstrates the costs and benefits of waiting for the current licensing agreement between PTA and APN to end^(41)^.

A$, Australian dollars in 2019 values; CBA, cost-benefit analysis; HALY, Health-adjusted life years; OOH, out of home; PTA, Public Transport Authority; TfL, Transport for London; UI, uncertainty intervals; UK, United Kingdom; VSLY, value of a statistical life year; WA, Western Australia.

#### Change in consumption of unhealthy food and drink, energy intake and health outcomes

To estimate the impact of the policy on daily kilojoule (kJ) intake, the adjusted effect size was applied to unhealthy food consumption in the Perth population aged > 2 years. Foods classified as unhealthy in the TfL analysis^(10)^ were matched to food categories using the Council of Australian Governments guidelines. The consumption of these foods in the Perth population by 5-year age and sex groups was extracted from the Australian Health Survey 2011–2012^(23)^ and the National Health Survey 2017–2018^(24)^. The energy content of these food products was estimated using the Australian Food, Supplement and Nutrient Database (AUSNUT) 2011–2013^(25)^.

In the primary analysis, the adjusted effect size from Yau *et al.* ^(10)^ was applied to the consumption of all unhealthy food and drink products as categorised by the Council of Australian Governments guidelines, including fast food consumption. Yau *et al.* ^(10)^ undertook additional analyses to assess spillover effects of the intervention on the purchasing of healthy products and found no significant changes in mean energy in the intervention group compared with the comparator. Therefore, substitution to more healthy foods was not included in this analysis. The Yau *et al.* ^(10)^ analysis also disaggregated the impact on purchasing by food category. They found that the change in energy of purchased food was significant for all unhealthy foods combined and the individual food categories of: (i) chocolate and confectionery and (ii) puddings and biscuits. In sensitivity analyses, the statistically significant effect sizes were applied to the relevant food categories (Scenario 5).

The change in daily kJ consumption was converted to changes in body weight using validated equations for children^(26)^ and adults and assumed to occur at the end of year 1^(27)^. A previously developed and validated proportional, multi-state lifetable Markov cohort model (ACE-Obesity Policy model)^(28–30)^, adapted for the 2019 Perth population,^(31)^ was used to estimate the impact of the changes in weight on body mass index (BMI) and the consequent impact on the epidemiology of nine obesity-related diseases (type 2 diabetes, hypertensive heart disease, ischaemic heart disease, stroke, osteoarthritis (hip and knee) and kidney, colorectal, endometrial and breast cancers). The long-term health outcomes were quantified as health-adjusted life years. Using cost data from the Australian Institute of Health and Welfare^(32)^, changes in healthcare costs resulting from changes in the incidence and prevalence of obesity-related diseases were also estimated.

For the cost–benefit analysis, the incremental health-adjusted life years gained from the impact of the policy were monetised using the value of a statistical life year (VSLY, A$213 000) as recommended by the Australian Government Office of Impact Analysis^(33)^. A more contemporary estimate of the VSLY (A$314 772 in 2019 values)^(34)^ was used in sensitivity analyses (Scenario 6).

### Cost of policy development and implementation

Using a societal perspective, cost to government included: policy development, policy monitoring and the impact of lost advertising revenue. Loss in profits was used as a proxy for loss of producer surplus for the OOH advertising industry. Loss of profits to unhealthy food and drink manufacturers from reduced sales was not included in the analysis, which is consistent with other cost–benefit analyses undertaken for government decision-making where it was assumed that a reduction in sales of unhealthy products will be redistributed to other unaffected products with minimal overall impact on economic output and the economy^(35)^. All wage rates were inclusive of 14 % salary oncosts^(36)^ and 17·5 % annual leave loading^(37)^.

#### Cost to government

The time required to develop the policy was informed by discussions with WA Government policymakers with experience in developing a policy to restrict OOH alcohol advertising. The primary analysis assumed that policy adoption would not require legislation. Sensitivity analyses (Scenario 7) tested the impact of including the cost of legislation ($1 306 180 in 2019 values)^(38)^ on the results.

Policy monitoring costs were estimated from economic evaluations of national television advertising restrictions^(36)^. These costs were halved for the current analysis given that a state-based policy would require less compliance monitoring compared to a nationwide policy. It was assumed that monitoring would require one full-time compliance officer.

Informed by parliamentary records^(39)^, potential advertising revenue loss to the WA government (from lower value contracts with advertising companies) was estimated from annual advertising income from food and drink advertising on PTA assets adjusted for the proportion of this advertising for unhealthy products (55 %)^(6)^. In the absence of data, the revenue loss for Main Roads WA was estimated based on industry reports indicating that 58 % of OOH advertising is on billboards^(40)^. The TfL experience showed no impact on advertising revenue after policy implementation, this is tested in Scenario 8^(12)^. The primary analysis used a conservative assumption that there was no replacement of the unhealthy food advertising and the loss in revenue was maintained over a 30-year time horizon. A less conservative assumption related to advertising replacement by products not impacted by the advertising restrictions was tested in Scenario 9.

A 10-year licensing agreement with PTA and APN (an outdoor advertising company) was signed in 2019^(41)^. Due to the lack of available data on contract details, the cost of early advertising contract termination was not included in the primary analysis. Scenario 10 models the impact of phasing in the intervention after 10 years when the licensing agreement expires.

#### Cost to industry

To estimate the potential revenue impact on the advertising industry, APN annual revenue from transport advertising across Australia^(40)^ was adjusted by: (i) advertising concentration in WA (7 %)^(42)^ and (ii) proportion of all advertisements that are for unhealthy food and drink (21 %)^(6)^. The profit margin for APN Outdoor group (13 %) was applied to the revenue to estimate potential profit losses if there were no advertising replacement (no impact on profits is tested in Scenario 8)^(43)^.

### Uncertainty analyses

Monte Carlo simulation using an Excel add-in software (Ersatz version 1.35) as undertaken to incorporate parameter uncertainty into all modelled outputs, which are reported with 95 % uncertainty intervals (UI). Drawing parameter input values defined by probability distributions (Table 1), 2000 iterations of the model were run. When there was minimal data on parameter distributions, the mean value was varied by ± 25 % using a Pert distribution^(44)^.

Uncertainty related to the assumptions used in the primary analysis was tested using univariate and multivariate scenario analyses. Various analyses were undertaken to evaluate the impact of changing individual variables or multiple varibles at once on the cost-effectiveness results. The scenarios examined included changes to the time horizon, costs and effectiveness and are described in Table 2.

See online supplementary material, Supplemental File 1 for the completed Consolidated Health Economic Evaluation Reporting Standards 2022 (CHEERS 2022) checklist for this economic evaluation.

## Results

### Primary analysis

Over a 30-year time horizon, a policy to restrict unhealthy food and drink advertisements on WA transport assets was estimated to result in change in weight of –0·58 kg (95 % UI: –0·90, –0·28) for the Greater Perth population. This translated to 5906 health-adjusted life years gained (95 % UI: 2750, 9084). The overall cost of intervention implementation was A$28 million (M) (95 % UI: $23, $35), with the majority (71 %) being borne by the WA Government. The majority (92 %) of benefits accrued from monetised health gains. However, intervention costs were offset by healthcare cost savings of A$116M (95 % UI: $56, $180), and therefore, even without the monetised health gains, the intervention would be considered dominant – producing health gains and net cost savings. The results demonstrated that the proposed policy is likely to result in substantial net societal benefits (Table 3; BCR: 50 (95 % UI: 23, 81); net present value: A$1346M (95 % UI: $614, $2082)).

**Table 3.** CBA results of primary analysis and scenarios 1 to 5 (mean, 95 % uncertainty interval (UI))

	Primary analysis results	Scenario 1	Scenario 2	Scenario 3	Scenario 4	Scenario 5
		10-year time horizon	4-year time horizon	A further 18 % reduction in the effect size	No adjustment to the effect size	Applying TfL statistically significant food categories only
Perth population change in body weight (kg)	–0·58 (–0·90, –0·28)	Same as primary analysis	Same as primary analysis	–0·46 (–0·70, –0·23)	–0·73 (–1·11, –0·36)	–0·47 (–0·75, –0·22)
Perth population change in daily energy intake (kJ)	–64·91 (–100·20, –31·52)	Same as primary analysis	Same as primary analysis	–50·76 (–78·14, –25·19)	–81·43 (–123·96, –39·74)	–52·24 (–83·21, –24·40)
Total HALYs gained	5906 (2750, 9084)	1215 (597, 1921)	168 (82, 256)	4676 (2305, 7314)	7365 (3416, 11 622)	5493 (2307, 8999)
Total intervention costs (millions)	$28 ($23, $35)	$16 ($13, $20)	$8 ($7, $10)	Same as primary analysis	Same as primary analysis	Same as primary analysis
% Government costs (millions)	$20 ($15, $26) 71 % of the total intervention cost	$12 ($9, $15) 72 % of the total intervention cost	$6 ($5, $8) 73 % of the total intervention cost	Same as primary analysis	Same as primary analysis	Same as primary analysis
% Industry costs (millions)	$8 ($7, $9) 29 % of the total intervention cost	$4 ($4, $5) 28 % of the total intervention cost	$2 ($2, $3) 27 % of the total intervention cost	Same as primary analysis	Same as primary analysis	Same as primary analysis
Total monetary benefits (millions)	$1374 ($642, $2112)	$288 ($142, $454)	$41 ($20, $62)	$1088 ($536, $1696)	$1713 ($799, $2695)	$1278 ($539, $2083)
Total HC savings (millions)	$116 ($56, $180) 8 % of the total monetary benefits	$29 ($14, $45) 10 % of the total monetary benefits	$5 ($2, $7) 12 % of the total monetary benefits	$92 ($43, $146) 8 % of the total monetary benefits	$145 ($66, $227) 8 % of the total monetary benefits	$108 ($44, $174) 8 % of the total monetary benefits
Value of health gains (millions)	$1258 ($586, $1935) 92 % of the total monetary benefits	$259 ($127, $409) 90 % of the total monetary benefits	$36 ($18, $55) 88 % of the total monetary benefits	$996 ($491, $1558) 92 % of the total monetary benefits	$1569 ($728, $2476) 92 % of the total monetary benefits	$1170 ($491, $1917) 92 % of the total monetary benefits
Net present value (NPV) (millions)	$1346 ($614, $2082)	$272 ($126, $439)	$33 ($11, $54)	$1060 ($509, $1670)	$1685 ($770, $2665)	$1250 ($509, $2056)
Benefit:cost ratio	50 (23, 81)	18 (9, 29)	5 (2, 8)	40 (19, 65)	62 (27, 104)	46 (19, 79)
Probability intervention has positive NPV	100 %	100 %	100 %	100 %	100 %	100 %

CBA, Cost–benefit analysis; HALY, Health-adjusted life year; HC, Healthcare; kg, Kilogram; kJ, Kilojoule; UI, Uncertainty intervals.

### Sensitivity analyses

Sensitivity analyses results are presented in Table 3 and 4. For the scenarios where the effectiveness parameters were changed, Scenario 10 (phasing in the intervention after 10 years to align with the end of current outdoor advertising contracts) produced the least favourable results (BCR 34 (95 % UI: 17, 56)). Applying the effectiveness of the TfL policy with no adjustments (Scenario 4) produced the most favourable results (BCR: 62 (95 % UI: 27, 104)).

Across all scenarios, assuming no change in advertising revenue to both advertisers and Government based on the TfL experience (Scenario 8) produced overall the most favourable outcomes (BCR: 808 (95 % UI: 400, 1299)). Using a very short time horizon of 4 years (Scenario 2) produced the least favourable results (BCR: 5 (95 % UI: 2, 8)).

## Discussion

This analysis found that a policy to restrict unhealthy food and drink advertising on WA state-owned public transport assets potentially represents excellent value for money. The policy is predicted to produce net benefits of almost A$1·4 billion over a 30-year period and produce A$50 in benefits for each dollar spent. The results of the sensitivity analyses demonstrated that under all tested scenarios, the policy was likely to produce net societal benefits.

These results are consistent with an evaluation of the TfL policy that used the same effectiveness measures (from Yau *et al.* 2022^(10)^) as the current analysis. It demonstrated that the TfL policy would result in substantial health gains and healthcare cost savings for the London population^(12)^. Although the London population is five times greater than the Perth population, the TfL evaluation predicted 3·8 times the health benefits compared with the current analysis. This could be explained by the current analysis modelling different and more diseases to estimate the long-term health impact of the policy. There are limited studies that report the cost-effectiveness of obesity prevention policies for the WA setting. A recent evaluation of a WA mass media campaign to encourage adults to achieve a healthy weight was reported to represent very good value for money^(31)^. This study adds to the evidence base of cost-effective actions that state governments can take to support healthy population diets.

A key concern of transport department policymakers may be the potential for reduced advertising revenue from lower-value advertising contracts with outdoor advertisers resulting from the policy. However, the experience from TfL was that there was no impact on TfL advertising revenue resulting from their advertising restrictions policy^(12)^, demonstrating the feasibility of replacement advertising on OOH assets. A study that evaluated industry responses to the TfL policy found that the strongest opposition to the TfL policy came from the advertising industry, claiming large revenue impacts^(45)^. This analysis shows that if there was no replacement of the unhealthy food advertisements, the potential profit loss to advertisers would be approximately A$8M over a 30-year period, with the more likely scenario of no impact on advertising industry profits. However, the cost of waiting for the 10-year OOH advertising contract to lapse was estimated to result in substantially less societal benefits.

A recent obesity prevention priority-setting study found that thirteen out of the sixteen interventions evaluated required multi-sectoral collaboration for effective implementation^(28)^. This analysis showed that the majority of intervention costs (A$20M) were likely to be borne by the transport department; however, the healthcare cost savings (A$116M) were accrued by state and federal departments of health. This highlights the need for structural changes to department financing to incentivise cross-sectoral collaboration in order to effectively implement public health policies that produce net societal benefits^(46)^.

There are several strengths of this analysis including the use of a validated economic model that has been used to evaluate several obesity prevention interventions for the Australian setting^(28)^. Another strength is the extensive involvement of an expert advisory group of policymakers and public health experts who confirmed the plausibility and policy relevance of the assumptions used and the scenarios tested. A key limitation was the inability to incorporate quantified productivity impacts of the policy (resulting from reduced chronic illness), which would have increased the societal benefits of the modelled policy. In addition, there were several key data gaps. Advertising exposure data was ascertained from industry reports that did not robustly report the data collection and analysis methods. Dietary survey data for the Perth population used in the modelling were dated, requiring the assumption that unhealthy food consumption patterns have remained relatively consistent over the past 10 years. Based on the evidence from Yau *et al.,* ^(10)^ dietary substitution of food and drink products that were subject to advertising restrictions to products that were not restricted was not modelled. If substitution exists, the impact of the policy on overall energy consumption is likely overestimated. Conversely, the health impacts of this policy may be underestimated in this analysis as the modelling exclusively used reductions in BMI to estimate the health gains associated with the policy. However, reduced consumption of unhealthy foods will also deliver health gains through reduced consumption of sugar, salt and saturated fat, which are all risk factors for chronic disease independent of BMI^(47)^. In addition, an important impact that is not included in the modelling is how advertising restrictions may shape perceptions of healthier eating patterns, potentially shifting social norms in ways that produce more substantial and lasting effects over time^(48)^. There were also several assumptions that were required to estimate the impact of this policy in the WA context including reliance of one study (Yau *et al*. 2022)^(10)^ that assessed the effectiveness of the TfL policy on unhealthy food purchasing by Londoners. Additional evaluations of outdoor unhealthy food and drink advertising restriction policies are required to strengthen the evidence base of the effectiveness of outdoor advertising restrictions and bridge current evidence gaps to inform future public health policy design including understanding i) substitution consumption of other products and overall impact on dietary intake and ii) response to the restrictions from unhealthy food and drink manufacturers and the advertising industry, including displacement of unhealthy food and drink advertising to unrestricted platforms and increasing master brand advertising alongside healthier products (which is currently permitted in the TfL policy).

Comprehensive advertising bans across all media are likely required to effectively reduce exposure to unhealthy food and drink advertising. The Australian Preventive Health Strategy 2021–2030^(49)^ and the National Obesity Strategy 2022–2032^(7)^ highlight the need to reduce exposure to unhealthy food and drink marketing. Recent progress has been made with the Australian Government Department of Health, Disability and Ageing undertaking a feasibility study on options to reduce unhealthy food marketing^(50)^. Whilst the federal government progresses these initiatives, Australian state governments can take a leading role by implementing this potentially highly cost-effective policy. Although the results of this analysis may not be directly transferable to other Australian states and territories due to differences in ownership and management of transport media assets and population characteristics, it provides evidence to support the development of a policy relevant to the context of different Australian jurisdictions. On 4 January 2025, another Australian State government (Government of South Australia) announced a new policy to restrict unhealthy food and drink advertising on buses and trains^(51)^. The South Australian governments should undertake a robust evaluation of its policy to add to the evidence of the effectiveness and cost-effectiveness of restricting outdoor unhealthy food and drink advertising. This may provide the necessary evidence for other Australian States and Territories to adopt a similar policy. National Government leadership may be required to ensure a consistent approach is adopted across the country.

### Conclusion

This study is the first full economic evaluation of a proposed policy to restrict unhealthy food and drink advertising on publicly owned outdoor assets. It demonstrates that such a policy is likely to represent excellent value-for-money and results in substantial societal benefits. This is a key strategy that Australian State governments can implement to protect their population from the harmful effects of unhealthy food and drink marketing.

**Table 4.** CBA results of primary analysis and scenarios 6 to 10 (mean, 95 % uncertainty interval (UI))

	Primary analysis results	Scenario 6	Scenario 7	Scenario 8	Scenario 9	Scenario 10
		A more contemporary estimate of the VSLY and lifetime time horizon	Cost of legislation	No change in gov and industry revenue	Government and industry revenue replacement/substitution	Phase in the intervention
Perth population change in body weight (kg)	–0·58 (–0·90, –0·28)	Same as primary analysis	Same as primary analysis	Same as primary analysis	Same as primary analysis	Same as primary analysis
Perth population change in daily energy intake (kJ)	–64·91 (–100·20, –31·52)	Same as primary analysis	Same as primary analysis	Same as primary analysis	Same as primary analysis	Same as primary analysis
Total HALYs gained	5906 (2750, 9084)	27 879 (13 861, 43 494)	5872 (2770, 9166)	5940 (2984, 9288)	5929 (2967, 9276)	2128 (1058, 3349)
Total intervention costs (millions)	$28 ($23, $35)	$67 ($54, $84)	$28 ($23, $35)	$2 ($1, $2)	$6 ($5, $8)	$15 ($12, $18)
% Government costs (millions)	$20 ($15, $26) 71 % of the total intervention cost	$48 ($35, $65) 71 % of the total intervention cost	$21 ($16, $27) 72 % of the total intervention cost	$2 ($1, $2) 100 % of the total intervention cost	$5 ($4, $6) 78 % of the total intervention cost	$11 ($8, $14) 72 % of the total intervention cost
% Industry costs (millions)	$8 ($7, $9) 29 % of the total intervention cost	$19 ($16, $23) 29 % of the total intervention cost	$8 ($7, $9) 28 % of the total intervention cost	$0 0 % of the total intervention cost	$1 ($1, $2) 22 % of the total intervention cost	$4 ($3, $5) 28 % of the total intervention cost
Total monetary benefits (millions)	$1374 ($642, $2112)	$9230 ($4593, $14 369)	$1366 ($644, $2124)	$1383 ($693, $2144)	$1379 ($688, $2156)	$496 ($246, $777)
Total HC savings (millions)	$116 ($56, $180) 8 % of the total monetary benefits	$454 ($223, $708) 5 % of the total monetary benefits	$116 ($54, $180) 8 % of the total monetary benefits	$118 ($59, $179) 9 % of the total monetary benefits	$116 ($57, $180) 8 % of the total monetary benefits	$43 ($22, $66) 9 % of the total monetary benefits
Value of health gains (millions)	$1258 ($586, $1935) 92 % of the total monetary benefits	$8776 ($4363, $13 691) 95 % of the total monetary benefits	$1251 ($590, $1952) 92 % of the total monetary benefits	$1265 ($636, $1978) 91 % of the total monetary benefits	$1263 ($632, $1976) 92 % of the total monetary benefits	$453 ($225, $713) 91 % of the total monetary benefits
Net Present Value (NPV) (millions)	$1346 ($614, $2082)	$9163 ($4516, $14 292)	$1338 ($615, $2097)	$1381 ($691, $2142)	$1373 ($682, $2150)	$482 ($230, $761)
Benefit cost ratio	50 (23, 81)	139 (64, 225)	49 (23, 79)	808 (400, 1299)	219 (107, 351)	34 (17, 56)
Probability intervention has positive NPV	100 %	100 %	100 %	100 %	100 %	100 %

CBA, Cost–benefit analysis; HALY, Health-adjusted life year; HC, Healthcare; kg, Kilogram; kJ, Kilojoule; NPV, Net present value; UI, Uncertainty Interval.

## Supporting information

Ananthapavan et al. supplementary material Ananthapavan et al. supplementary material

## Supplementary material

For supplementary material accompanying this paper visit https://doi.org/10.1017/S1368980026102249
