# Supplementary material for: Potential cost-effectiveness of outdoor unhealthy food and drink advertising restrictions in Western Australia
Source: Public Health Nutr. 2026 Feb 26;29(1):e54. doi: 10.1017/S1368980026102249 (PMC13112306; doi:10.1017/S1368980026102249)
Supplement: Ananthapavan et al. supplementary material [file S1368980026102249sup001.doc]

# **Supplementary files**

Supplementary file 1 CHEERS 2022 Checklist

| Topic | No. | Item | Location where item is reported |
| --- | --- | --- | --- |
| Title |  |  |  |
|  | 1 | Identify the study as an economic evaluation and specify the interventions being compared. | Title page  Abstract |
| Abstract |  |  |  |
|  | 2 | Provide a structured summary that highlights context, key methods, results, and alternative analyses. | Abstract |
| Introduction |  |  |  |
| Background and objectives | 3 | Give the context for the study, the study question, and its practical relevance for decision making in policy or practice. | Introduction |
| Methods |  |  |  |
| Health economic analysis plan | 4 | Indicate whether a health economic analysis plan was developed and where available. | Economic analysis plan was not developed |
| Study population | 5 | Describe characteristics of the study population (such as age range, demographics, socioeconomic, or clinical characteristics). | Methods, under “Policy impact on population weight and BMI” ( |
| Setting and location | 6 | Provide relevant contextual information that may influence findings. | Methods, under “The proposed policy” and “2.3 Policy impact on population weight and BMI” |
| Comparators | 7 | Describe the interventions or strategies being compared and why chosen. | Methods, under “Overview of the economic evaluation” |
| Perspective | 8 | State the perspective(s) adopted by the study and why chosen. | Methods, under “Overview of the economic evaluation” |
| Time horizon | 9 | State the time horizon for the study and why appropriate. | Methods, under “Overview of the economic evaluation” |
| Discount rate | 10 | Report the discount rate(s) and reason chosen. | Methods, under “Overview of the economic evaluation” |
| Selection of outcomes | 11 | Describe what outcomes were used as the measure(s) of benefit(s) and harm(s). | Methods, under “Policy impact on population weight and BMI” |
| Measurement of outcomes | 12 | Describe how outcomes used to capture benefit(s) and harm(s) were measured. | Methods, under “Policy impact on population weight and BMI” |
| Valuation of outcomes | 13 | Describe the population and methods used to measure and value outcomes. | Methods, under “Policy impact on population weight and BMI” |
| Measurement and valuation of resources and costs | 14 | Describe how costs were valued. | Methods, under “Cost of policy development and implementation” |
| Currency, price date, and conversion | 15 | Report the dates of the estimated resource quantities and unit costs, plus the currency and year of conversion. | Table 1-2 and Supplementary files 2-4 |
| Rationale and description of model | 16 | If modelling is used, describe in detail and why used. Report if the model is publicly available and where it can be accessed. | Methods, under subheading "Change in consumption of unhealthy food and drink, energy and health outcomes" |
| Analytics and assumptions | 17 | Describe any methods for analysing or statistically transforming data, any extrapolation methods, and approaches for validating any model used. | Not applicable |
| Characterising heterogeneity | 18 | Describe any methods used for estimating how the results of the study vary for subgroups. | Not applicable |
| Characterising distributional effects | 19 | Describe how impacts are distributed across different individuals or adjustments made to reflect priority populations. | Not applicable |
| Characterising uncertainty | 20 | Describe methods to characterise any sources of uncertainty in the analysis. | Methods, under "Uncertainty analyses" |
| Approach to engagement with patients and others affected by the study | 21 | Describe any approaches to engage patients or service recipients, the general public, communities, or stakeholders (such as clinicians or payers) in the design of the study. | Methods, under "Effectiveness adjustments", pages 6-7, and acknowledgement |
| Results |  |  |  |
| Study parameters | 22 | Report all analytic inputs (such as values, ranges, references) including uncertainty or distributional assumptions. | Result section and tables 3-4 |
| Summary of main results | 23 | Report the mean values for the main categories of costs and outcomes of interest and summarise them in the most appropriate overall measure. | Result section and tables 3-4 |
| Effect of uncertainty | 24 | Describe how uncertainty about analytic judgments, inputs, or projections affect findings. Report the effect of choice of discount rate and time horizon, if applicable. | Result section particularly under scenario analyses and tables 3-4 |
| Effect of engagement with patients and others affected by the study | 25 | Report on any difference patient/service recipient, general public, community, or stakeholder involvement made to the approach or findings of the study | Not applicable |
| Discussion |  |  |  |
| Study findings, limitations, generalisability, and current knowledge | 26 | Report key findings, limitations, ethical or equity considerations not captured, and how these could affect patients, policy, or practice. | Discussions section |
| Other relevant information |  |  |  |
| Source of funding | 27 | Describe how the study was funded and any role of the funder in the identification, design, conduct, and reporting of the analysis | Title page |
| Conflicts of interest | 28 | Report authors conflicts of interest according to journal or International Committee of Medical Journal Editors requirements. | Title page |

*Source^64^:* Husereau D, Drummond M, Augustovski F, et al. Consolidated Health Economic Evaluation Reporting Standards 2022 (CHEERS 2022) Explanation and Elaboration: A Report of the ISPOR CHEERS II Good Practices Task Force. Value Health 2022;25. <doi:10.1016/j.jval.2021.10.008>

Supplementary file 2 Key inputs used in the Primary analyses and Scenarios 1 to 5

| **Key Parameters** | **Primary Analysis** | **Scenario 1** | **Scenario 2** | **Scenario 3** | **Scenario 4** | **Scenario 5** |
| --- | --- | --- | --- | --- | --- | --- |
| Intervention duration/Model time horizon | 30 years | 10 years | 4 years | Same as primary analysis | Same as primary analysis | Same as primary analysis |
| Discount rate | 7% | Same as primary analysis | Same as primary analysis | Same as primary analysis | Same as primary analysis | Same as primary analysis |
| Intervention effectiveness adjustments | -5.36% (95%UI: -2.64 to -8.14) | Same as primary analysis | Same as primary analysis | -4.2% (95% UI: -2.1 to -6.3) | -6.7% (95% UI: -10.1 to -3.2) | Effect size:  Chocolate & Confectionary: -15.6% (95% UI: -11.0% to -20.4%)  Puddings & Biscuits: -5.2% (95% UI: 0.4% to 10.0%) |
| Food categories included | All consumption except diet drinks using all total HFSS effect size | Same as primary analysis | Same as primary analysis | Same as primary analysis | Same as primary analysis | Effect size applied to respective food products |
| Estimated cost of passing a policy | A$755,915 (95% UI: 533,455 to 1,011,806)  (one-time cost) | Same as primary analysis | Same as primary analysis | Same as primary analysis | Same as primary analysis | Same as primary analysis |
| Estimated revenue loss to PTA and Main Roads WA | Total revenue loss (PTA and Mains Road WA): A$1,381,748 per year (95%UI: 994,945 to 1,899,238) | Same as primary analysis | Same as primary analysis | Same as primary analysis | Same as primary analysis | Same as primary analysis |
| Estimated cost of monitoring the policy | A$73,060 per year (95% UI: 59,226 to 86,832) | Same as primary analysis | Same as primary analysis | Same as primary analysis | Same as primary analysis | Same as primary analysis |
| Potential impact on advertising industry profits | A$596,313 per year (95% UI: 494,034 to 704,306) | Same as primary analysis | Same as primary analysis | Same as primary analysis | Same as primary analysis | Same as primary analysis |
| Monetised health gains (VSYL) | A$213,000 per HALY | Same as primary analysis | Same as primary analysis | Same as primary analysis | Same as primary analysis | Same as primary analysis |

Notes: HFSS: High-fat, salt and sugar products; PTA: Public Transport Authority; UI: Uncertainty Intervals; VSLY: Value of a statistical life year

Supplementary file 3: Key inputs used in Scenarios 6 to 10

| **Key Parameters** | **Scenario 6** | **Scenario 7** | **Scenario 8** | **Scenario 9** | **Scenario 10** |
| --- | --- | --- | --- | --- | --- |
| Intervention duration/Model time horizon | Lifetime | Same as primary analysis | Same as primary analysis | Same as primary analysis | Same as primary analysis |
| Discount rate | 3% | Same as primary analysis | Same as primary analysis | Same as primary analysis | Same as primary analysis |
| Intervention effectiveness adjustments | Same as primary analysis | Same as primary analysis | Same as primary analysis | Same as primary analysis | Phase in the intervention in 10 years when the contract ends. |
| Food categories included | Same as primary analysis | Same as primary analysis | Same as primary analysis | Same as primary analysis | Same as primary analysis |
| Estimated cost of passing a policy | Same as primary analysis | Cost of legislation in 2019: $1,302,830 (95%UI: $1,129,184 to $1,494,272) | Same as primary analysis | Same as primary analysis | Same as primary analysis |
| Estimated revenue loss to PTA and Main Roads | Same as primary analysis | Same as primary analysis | No change in government ad revenue | **Total discounted government revenue: A$3,212,026 (95% UI: 2,343,318 to 4,403,638) | Phase in the intervention in 10 years when the contract ends. |
| Estimated cost of monitoring the policy | Same as primary analysis | Same as primary analysis | Same as primary analysis | Same as primary analysis | Phase in the intervention in 10 years when the contract ends. |
| Potential impact on advertising industry profits | Same as primary analysis | Same as primary analysis | No change in advertiser ad revenue | **Total discounted industry revenue loss*: A$1,398,106 (95% UI: 1,149,250 to 1,647,095) | Phase in the intervention in 10 years when the contract ends. |
| Monetised health gains (VSYL) | VSLY valued at A$314,772 | Same as primary analysis | Same as primary analysis | Same as primary analysis | Same as primary analysis |

Notes: *Estimated by adding and discounting revenue losses as follows: Year 1 at 100%, Year 2 at 75%, Year 3 at 50%, Year 4 at 25%, and Year 5 onwards at 0%; HFSS: High-fat, salt and sugar products; PTA: Public Transport Authority; UI: Uncertainty Intervals; VSLY: Value of a statistical life year

Supplementary file 4 Cost parameters

| **Cost parameter** | **Analysis** | **Cost in 2019 (mean)** | **Distribution used in uncertainty analysis** |
| --- | --- | --- | --- |
| Cost of passing a policy | Primary analysis | A$755,915 (95% UI: 533,455 to 1,011,806)  (one-time cost) | 2.4 FTE for 12 months – based on advice from WA policymakers.  Unit cost A$163 per hour in 2021/22 value ^71^ adjusted to 2019 value.  The unit cost, number of hours spent implementing the policy and the number of FTE were varied by ±25% and sampled using a Pert distribution. |
|  | Sensitivity analysis: cost of passing legislation (scenario 7) | A$1,306,180  (SE: A$92,867) (one-time cost) | A$1,090,000 (SE: A$77,497.00) ^53^ in 2010 value inflated to 2019 value  Gamma distribution |
| Changes in advertising revenue to government (PTA and Main Roads) | Primary analysis | A$1,381,748 per year (95%UI: 994,945 to 1,899,238) | PTA revenue from food and drink advertising ^55^:  2017/18 A$1,006,050 and 2018/19 A$1,002,984  Proportion of food advertising that is for unhealthy foods: 55% ^10, 11^  Main roads WA share of advertising revenue: 58% ^56^  The proportion of food advertising that is for unhealthy foods and Main roads WA share of advertising revenue were varied using a Pert distribution with a mean of ±25% |
|  | Sensitivity analysis: no revenue loss  (scenario 8) | A$0 | Not applicable |
|  | Sensitivity analysis (revenue loss year 1: 100%, year 2: 75%, year 3: 50%, year 4: 25%, year 5 onwards: 0%) (scenario 9) | **Total discounted government revenue**: A$3,212,026 (95% UI: 2,343,318 to 4,403,638)  **Mean cost by year**  Year 1: A$1,350,757  Year 2: A$1,013,068  Year 3: A$675,378  Year 4: A$337,689  Year 5 onwards: A$0 | Same as primary analysis |
| Cost of monitoring the policy | Primary analysis | A$73,060 per year (95% UI: 59,226 to 86,832) | Weekly wage of personnel for legislation administration: A$1,258 (SE: 41.5) in 2021 value adjusted to 2019 value ^72^  Labour on-cost (14% salary cost ^72^): $8,849 in 2019 value  Annual leave loading (17.5% weekly salary cost, 4 weeks per annum ^51^): $851 in 2019 value  Assumption: 1.0 FTE administration and compliance officer.  Weekly wage of personnel for legislation administration was varied using Gamma distribution. Salary on-cost, annual leave loading salary costs and the number of FTEs required for this task were varied using a Pert distribution with a mean of ±25% |
| Penalty for early termination of contract | Primary analysis | Not included | Not applicable |
|  | Sensitivity analysis (Phase in the intervention 10 years after the contract)  (scenario 10) | Impact on government revenue and industry profits accrue after 10 years. | Same as primary analysis |
| Impact on advertiser profits | Primary Analysis | A$596,313 per year (95% UI: 494,034 to 704,306) | APN revenue from transport assets: A$302.3M in 2017 values ^56^ adjusted to 2019 values and adjusted by advertising concentration in WA (7% ^60^), proportion of discretionary ads over all ads (21% ^10, 11^) and a 13% profit margin ^61^.  The proportion of discretionary ads over all ads was varied using a Pert distribution with a mean of ±25% |
|  | Sensitivity analysis: no revenue loss  (scenario 8) | A$0 | Not applicable |
|  | Sensitivity analysis: (revenue loss year 1: 100%, year 2: 75%, year 3: 50%, year 4: 25%, year 5 onwards: 0%)  (scenario 9) | Total discounted industry revenue loss: A$1,398,106 (95% UI: 1,149,250 to 1,647,095)  **Mean cost by year**  Year 1: A$596,993  Year 2: A$447,745  Year 3: A$298,496  Year 4: A$149,248  Year 5 onwards: A$0 | Same as primary analysis |

Notes: UI: Uncertainty Intervals; PTA: Public Transport Authority; FTE: Full time equivalent; WA: Western Australia; SE: Standard error
